# 

**Published:** 1921-08-27

**Authors:** 


### An Attractive Programme.

The Sanitary Inspectors' Association will hold its thirty-fourth Annual Conference at Bath from August 30 to September 2. We congratulate the Conference Committee on the form of its programme, which is attractively got up, with illustrations of Bath, a well-printed map, and several blank pages of good quality lined paper for notes, each page being perforated.

[We regret that, owing to pressure on our space, we have had to hold over some of our regular features, including correspondence.]

### TEACHING INSTITUTIONS IN DENTISTRY.

Particulars of Dental Schools connected with London hospitals are given on the previous pages. Provincially there are the Birmingham Dental Hospital, Leeds University School of Dentistry, the Dental Hospital of Manchester, Newcastle-upon-Tyne Dental Hospital and School, and the Devon and Exeter Dental Hospital. In Edinburgh the Dental Hospital and School is located in a spacious and well-equipped building at 31 Chambers Street, and offers special advantages to dental students. At Glasgow Dental Hospital the school is open to men and women, and the Glasgow Royal Infirmary has attached to it an excellent dental department. Complete training in the dental subjects is provided in Ireland at the Dental Hospital, Lincoln Place, Dublin, the medical portions being taught at the three medical schools.

August 27, 1921] [Price Twopence

### The Hospital

**RATES OF SUBSCRIPTION** (post free, payable in advance).

**Three months, 3/3; six months, 6/6; twelve months, 13/-.** (Should the cost of printing and paper as well as increased postage, necessitate alteration of these rates, the period paid for will be reduced proportionately on unexpired subscriptions.) **THE HOSPITAL "Index" to Volume LXIX., October 1920 to March 1921, is now ready, price 1/1 per copy, post free.** Address The Manager, THE HOSPITAL, "The Hospital" Building, 28/29 Southampton Street, Strand, London, W.C. 2.

### Publications.

Third Edition. Demy 8vo. 248 pages. Price **12s. 6d.** net.

**THE TREATMENT OF DISEASES OF THE SKIN.**

By W. KNOWSLEY SIBLEY, M.A., M.D., B.C. (Camb.), M.R.C.P., Physician to St. John's Hospital for Diseases of the Skin, London. With 39 Original Photographs and 156 Special Prescriptions

LANCET.—"Within recent years many new methods of treatment of the diseases of the skin have been introduced, and as a result the treatment of many of these affections has been revolutionised. Electrolysis, the X Rays, cataphoresis and currents of high frequency are some of the most striking of these new therapeutic agents; but many others are also of importance, such as radium and carbonic acid snow. Dr. Knowsley Sibley has included in his little work on the treatment of the diseases of the skin all these new methods of treatment. The book strikes us as well suited for the purpose of enabling the reader to see within a small compass the most modern means of dealing with each skin affection. . . . We consider the author has succeeded in what his title promises."

LONDON: EDWARD ARNOLD, 41 & 43 MADDOX STREET, W.

A MANUAL OF

**MEDICAL EXERCISES.**

By PERCY LEWIS, M.D., M.R.C.S.,

Hon. Medical Officer to Victoria Hospital, Folkestone.

66 pages, with 35 Illustrations, cloth, **2s. 6d.** net; post free, **2s. 7d.**

**H. K. LEWIS & CO., Ltd., 136 GOWER ST., LONDON, W.C.**

THIRD EDITION, Pp. viii.+92. With 4 Plates. Price 4s. 6d. net, postage 4d

**THE INTENSIVE TREATMENT OF SYPHILIS AND LOCOMOTOR ATAXIA BY AACHEN METHODS.**

By REGINALD HAYES, M.R.C.S.

THE LANCET says: "No better exposition of the method as practised there can be found than that contained in this little book."

Baillière, Tindall & Cox, 8 Henrietta Street, London, W.C. 2.

SECOND EDITION, in Two Vols. 45s. net.

**DEFORMITIES,**

INCLUDING

**DISEASES of the BONES and JOINTS.**

A Text-Book of Orthopædic Surgery.

Illustrated by 70 Plates and over 1,000 Figures and by Notes of Cases.

By **A. H. TUBBY, C.B., C.M.G., M.S.**

"The standard text-book in English on the subject."—*Lancet.*

First Edition. "Standard work on the subject in the English language." *British Medical Journal.*

**MACMILLAN & CO., LTD., St. Martin Street, London, W.C.**

**INDISPENSABLE TO THE GENERAL PRACTITIONER**

**"THE PRESCRIBER"**

**A MONTHLY JOURNAL DEALING WITH TREATMENT**

Keeps the Physician posted each month in all new Developments.

20*s. per annum, post free. Single Copies* 2*s., by post* 2*s.* 3*d.*

*Specimen copy free on application.*

**"THE PRESCRIBER" Offices, 6 South Charlotte Street, Edinburgh.**

**DELICATE CHILDREN: THEIR CARE AND MANAGEMENT.**

*By DR. PERCY LEWIS.*

Cheap Edition, in limp cloth binding, 1/6 net; by post, 1/9.

**THE SCIENTIFIC PRESS, LIMITED, 28/29 Southampton Street, Strand, London, W.C.**

**THE ELEMENTS OF PRACTICAL PSYCHO-ANALYSIS.**

By PAUL BOUSFIELD M.R.C.S., L.R.C.P.,

Physician to the London Neurological Clinic.

With Glossary and one Plate. **10s. 6d.** net.

"A more able, clear and concise exposition of this difficult subject would be hard to imagine."—*Medical Times.*

KEGAN PAUL & CO., Ltd., 68-74 Carter Lane, London, E.C.

**Publications**—*continued.*

### J. & A. CHURCHILL

### GASK and WILSON'S SURGERY.

**A Text-Book by Various Authors.**

39 Plates, 20 in Colour, 467 Text Figures. £2 2s. net. Postage 1s. 3d. Edited by G. E. GASK, C.M.G., D.S.O., F.R.C.S., and HAROLD W. WILSON, M.S., F.R.C.S., Surgeons to St. Bartholomew's Hospital.

"*The book is good, not too big, well printed and illustrated, and worthy of the school from which it comes.*" British Medical Journal.

"*The book is a notable addition to our surgical literature.*"—British Journal of Surgery.

### EDEN and LOCKYER'S GYNAECOLOGY.

2nd Edition. 24 Coloured Plates, 513 Text Figures. 42s. net. Postage 1s. 3d. By T. W. EDEN, M.D., F.R.C.P., F.R.C.S., & CUTHBERT LOCKYER, M.D., F.R.C.P., F.R.C.S., Obstetric Physicians, Charing Cross Hospital.

"*In every way the new edition maintains the high standard of its predecessor, and it will continue to rank as probably the most satisfactory all-round text-book of moderate size on the subject published in this country.*"—British Medical Journal.

**Collis and Greenwood's Health of the Industrial Worker.** With Special Chapter on Reclamation of the Disabled. With an Introduction by Sir GEORGE NEWMAN, K.C.B., D.C.L. With 38 Illustrations. 30s. net. Postage 1s.

"*This is the first adequate modern treatise upon the hygiene of industry in general. A more ideal combination of authors for this purpose it would be difficult to find. The book reaches an exceptionally high standard.*"—C. S. Myers, F.R.S., in Nature, May 19, 1921.

**Small's Text-Book of Botany.** For Medical and Pharmaceutical Students. With 1,350 Illustrations. 25s. net. Postage 1s.

**Bowlby and Andrewes' Surgical Pathology.** 7th Edition. With 200 New Illustrations. 30s. net. Postage 1s.

**Jex-Blake's Physical Signs in the Chest and Abdomen.** 27 Illustrations. 9s. 6d. net. Postage 4d.

**Craig's Psychological Medicine.** 3rd Edition. 27 Plates. 18s. net. Postage 6d.

**Berkeley & Bonney's Difficulties and Emergencies of Obstetric Practice.** 3rd Edition. 309 Illustrations. 42s. net. Postage 1s.

**Cushny's Pharmacology.** 7th Edition. 71 Illustrations. 24s. net. Postage 1s.

**Sequeira's Diseases of the Skin.** 3rd Edition. 52 Coloured Plates, 257 Text Figures. 38s. net. Postage 1s. 3d.

**Lee's Microtomists' Vade - Mecum.** 8th Edition. Revised and Edited by Dr. J. BRONTË GATENBY. 28s. net. Postage 1s.

**Marshall's Microbiology for Agricultural and Domestic Science Students.** 3rd Edition. 21s. net. Postage 1s.

**Coles' Critical Microscopy.** How to get the Best out of the Microscope. 8 Illustrations. 7s. 6d. net. Postage 5d.

**Orla - Jensen's Dairy Bacteriology.** Translated by P. S. ARUP, B.Sc., F.I.C. 70 Illustrations. 18s. net. Postage 6d.

**Eden's Manual of Midwifery.** 5th Edition. 5 Plates (4 in Colour), 269 Text Figures. 24s. net. Postage 1s.

"*We have nothing but praise for this book.*"—The Lancet.

**Taylor's Principles and Practice of Medical Jurisprudence.** New Edition. Edited by FRED. J. SMITH, M.D., F.R.C.P. 7th Edition. With 20 Engravings. Two Vols. £3 3s. net. Postage 1s. 3d.

"*For the lawyer as well as the medical practitioner this standard work is invaluable. In every branch of law into which medical considerations enter it may always be consulted with profit.*"—The Justice of the Peace.

**Arvedson's Notes on Diseases treated by Medical Gymnastics and Massage.** Translated by MINA L. DOBBIE, M.D. 8s. 6d. net. Postage 4d.

**Kleen's Massage and Medical Gymnastics.** Translated by MINA L. DOBBIE, M.D. B.Ch., Med. Officer, Chelsea Phys. Training Coll. 2nd Edition. 182 Illustrations. 32s. net. Postage 1s.

"*The book will be found a most valuable contribution to physico-therapeutic literature. It is admirably produced.*" The Lancet.

**Mennell's Massage: Its Principles and Practice.** 2nd Edition. 167 Illustrations. 21s. net. Postage 1s.

**Fleming's Medicine.** 3rd Edition. 64 Illustrations. 21s. net. Postage 1s.

**Parsons' Diseases of the Eye.** 3rd Edition. 18 Plates. 319 Text Figures. 18s. net. Postage 1s.

**Hale-White's Materia Medica.** 17th Edition. 10s. 6d. net. Postage 5d.

### *Colloids.*

**Hatschek's Laboratory Manual of Elementary Colloid Chemistry.** 20 Illustrations. 7s. 6d. net. Postage 3d.

***By the same Author.***

**Introduction to the Physics and Chemistry of Colloids.** 3rd Edition. 17 Illustrations. 5s. net. Postage 3d.

**Ostwald's Handbook of Colloid Chemistry.** 93 Illustrations. 18s. net. Postage 9d.

**Svedberg's Formation of Colloids.** 22 Illustrations. 7s. 6d. net. Postage 3d.

**London: J. & A. CHURCHILL, 7 Great Marlborough St., W.1.**

